# Whole-genome characterization and phylogenetic analysis of pigeon circovirus in racing pigeons from Heilongjiang, China

**DOI:** 10.3389/fvets.2025.1685178

**Published:** 2026-01-05

**Authors:** Jia Sun, Yan Miao, Shijie Lan, Jingjing Zhao, Wenying Dong, Jia Hu, Hansong Li, Huinan Li, Xinran Li, Ming Wang, Yaru Xu, Chunqiu Li, Xiaoxu Xing, Dan Yang, Qinghe Zhu

**Affiliations:** 1 College of Animal Science and Veterinary Medicine, Heilongjiang Bayi Agricultural University, Daqing, China; 2 Branch of Animal Husbandry and Veterinary of Heilongjiang Academy of Agricultural Sciences, Qiqihar, China

**Keywords:** pigeon circovirus, whole-genome sequence, capsid protein, recombination, evolution

## Abstract

Pigeon circovirus (PiCV), a globally distributed pathogen, is associated with immunosuppression and high mortality in racing pigeons. Despite its endemicity in Chinese pigeon populations, molecular epidemiological data on PiCV in Northeast China remain scarce. This study reports the first complete genome sequence of PiCV isolated from racing pigeons in Heilongjiang Province. Through next-generation sequencing (NGS) for whole-genome assembly and systematic PCR-Sanger sequencing for validation, we obtained the complete 2,035-bp genome (designated HLJ2024). Homology analysis revealed nucleotide identities of 72.9–97.7% with other PiCV reference strains; notably, the cap (Capsid) protein of HLJ2024 exhibited a signature mutation (isoleucine [I]-leucine [L]) at residue 222 relative to its closest relative strain TF4/SN/2016, with predicted structural alterations proximal to this site. Phylogenetic analysis indicated that strain HLJ2024 forms an independent clade (GI) and is closely related to strains of clade GII (TF4/SN/2016, QD6/SN/2018). Recombination analysis suggested that HLJ2024 likely arose from recombination between two Chinese parental strains-GF17/GuangDong/2014 (major parent) and TY2/SN/2016 (minor parent). Our findings reveal active PiCV evolution in Northern China driven by point mutations and inter-regional recombination, providing critical insights into viral adaptability and transmission dynamics in Northeast China. These results provide a foundation for molecular epidemiology-based surveillance of PiCV in this understudied region and hold significant implications for developing targeted prevention strategies tailored to locally circulating strains.

## Introduction

Pigeon circovirus (PiCV), the primary etiological agent of young pigeon disease syndrome (YPDS), is characterized by bursal atrophy, thymic lymphocyte apoptosis, and multi-organ lymphoid depletion, causing mortality rates of up to 60–70% in juvenile pigeons (1, 2). As a non-enveloped single-stranded DNA (ssDNA) virus (~20 nm diameter) within the Circoviridae family, PiCV shares key taxonomic features with duck circovirus (DuCV), beak and feather disease virus (BFDV), and porcine circovirus (PCV) (3). The PiCV genome (~2.0 kb) contains bidirectional open reading frames (ORFs): ORF-V1 encodes the replication-associated protein (Rep), which governs rolling-circle replication, while ORF-C1 encodes the Capsid protein (Cap). The Cap protein self-assembles into icosahedral virions and serves as the principal immunogen, mediating cell entry and eliciting neutralizing antibody responses (4). However, significant inter-genotypic sequence variation within the Cap protein drives antigenic divergence, severely compromising vaccine efficacy (5, 6).

Heilongjiang Province, located in Northeast China and a key region for racing pigeon breeding, suffers substantial economic losses due to recurrent PiCV outbreaks (7–9). While molecular characteristics of PiCV strains have been documented in provinces such as Guangdong and Shandong (10), the genetic evolution of Heilongjiang strains—particularly the dynamics of the Cap gene—remains largely uncharacterized.

This study integrates whole-genome phylogenetic analysis, Cap structural modeling analysis, and recombination detection analysis to decipher the adaptive mutation trajectories of PiCV strains in Heilongjiang, reveal key mutations in the capsid protein and their potential functional implications, and lay a foundation for targeted vaccine development. This research aims to provide molecular evidence for elucidating the genetic evolutionary mechanisms of PiCV, offer insights for formulating precise prevention and control strategies, and emphasize the necessity of systematic surveillance of PiCV evolutionary dynamics.

## Materials and methods

### Sample collection and processing

In July 2024, a PiCV outbreak occurred at a racing pigeon farm in Qiqihar, Heilongjiang Province, China, resulting in the death of 22 pigeons. Among these 22 deceased pigeons, a total of 6 PiCV-positive samples were detected through PCR screening. For the present study, one representative positive sample was selected for whole-genome sequencing and analysis.

### PCR identification and sequencing

To confirm the positivity of the samples prepared for sequencing via PCR, PiCV-positive liver samples were homogenized and mixed with phosphate-buffered saline (PBS) at a ratio of 1:3 (w/v) using a vortex mixer, after which the mixture was centrifuged at 1500 × g for 10 min; following centrifugation, the supernatant was collected for DNA extraction, which was performed as described previously (10–12). Conventional PCR was carried out using a one-step PCR kit (TaKaRa, Dalian, China) to amplify the partial sequence (678 bp) of PiCV with specific primers (PiCV-F: 5’-ACCTTGAACAACCCCACCGA-3′, PiCV-R: 5’-GCAGGAATGCCCAGGGTAAG-3′); a 25 μL reaction system consisted of 12.5 μL of 2 × one-step PCR buffer, 1 μL each of forward and reverse primers (10 μmol/L), 2 μL of DNA template, and 8.5 μL of nuclease-free water, and cycling conditions included initial denaturation at 94 °C for 5 min, followed by 35 cycles of denaturation at 94 °C for 30 s, annealing at 58 °C for 30 s, extension at 72 °C for 1 min, and final extension at 72 °C for 10 min before storage at 4 °C; and the products of this PCR assay were sent to Sangon biotech (Shanghai) co., ltd. for the detection of Newcastle disease virus (NDV), primers (NDV-F: 5’-GGGCTCCAAGGCTCCAAAGGAT-3′, NDV-R: 5’-CTTCCCAACTGCCACTGATAGT-3′) were used, targeting a 541-bp fragment (Supplementary Figure S1).

### Next-generation sequencing

Positive PiCV DNA was obtained as previously described. Illumina sequencing and library construction were performed at Shanghai Tanpu Biotechnology Co., Ltd. (Shanghai, China). Briefly, viral DNA was fragmented with NEBNext® dsDNA Fragmentase®. The fragmented DNA was used as input for library preparation following the protocol of the NEBNext® Ultra™II DNA Library Prep Kit for Illumina®. Library quality and concentration were quantified using an Agilent 2,100 Bioanalyzer with a High Sensitivity DNA Kit. Sequencing was conducted on an Illumina NovaSeq 6,000 platform following the manufacturer’s protocol, generating paired-end 150 nt reads.

### Post-sequencing data processing and quality control

Raw sequencing reads were subjected to quality control using fastp v0.20.0 software to filter out low-quality reads (reads with adapter contamination, ambiguous bases > 5%, or Phred quality score < 20). After filtering, 58,149,342 valid reads were retained with a Q30 score of 92.99%. Subsequently, the BBmap v38.51 software was employed to remove rRNA and host genomic DNA sequences from the valid reads to minimize non-target sequence interference. A total of 4,420,266 cleaned reads were finally obtained and used for subsequent species annotation and *de novo* sequence assembly.

### Verification of NGS-derived sequences by PCR-sanger sequencing

To validate the accuracy of the NGS-assembled PiCV sequence and correct potential false-positive variants, PCR-Sanger sequencing (the gold standard for sequence verification) was performed. Specific overlapping primers covering the full-length PiCV genome were designed based on the NGS-assembled sequence (primer sequences are provided in Supplementary Table S1). PCR amplification was carried out using PrimeSTAR Max DNA Polymerase (TaKaRa, Dalian, China) under the following conditions: initial denaturation at 94 °C for 5 min; 35 cycles of denaturation at 94 °C for 30 s, annealing at primer-specific temperatures (55–58 °C) for 30 s, and extension at 72 °C for 2 min; and final extension at 72 °C for 10 min. PCR products were separated by 1% agarose gel electrophoresis, purified using a Gel Extraction Kit (Omega Bio-tek, United States; Supplementary Figure S2), and subjected to bidirectional Sanger sequencing by Sangon Biotech (Shanghai, China).

Sequencing electropherograms were analyzed using Chromas Pro 2.6.6 software, and the obtained sequences were assembled into the full-length genome using DNAMAN 9.0. Alignment of the Sanger-assembled sequence with the NGS-derived sequence was performed using ClustalW (MEGA 11) to identify discrepancies. Discrepant sites were confirmed by three independent PCR-Sanger sequencing replicates, and only variants with consistent results were considered reliable. The final PiCV sequence used for subsequent analyses was determined based on the verified Sanger sequencing results, and the updated sequence has been submitted to GenBank.

### Sequence analysis

The viral open-reading frames (ORFs) prediction, amino acid (aa) translation, sequence alignment, and pairwise sequence comparisons were performed using the modules of EditSeq and MegAlign in DNASTAR Lasergene 12 Core Suite (DNASTAR, Inc., Madison, WI, United States). The highest similarities between the studied PiCV genome and the published sequences were identified by the BLASTN online search tool.^1^ Multiple sequence alignments were performed using the multiple sequence alignment tool in the DNAMAN 6.0 software (Lynnon BioSoft, Point-Claire, Quebec, Canada).

### Phylogenetic analysis

For the phylogenetic analysis, reference sequences of PiCV genes were retrieved from the NCBI nucleotide database (Supplementary Table S2). Phylogenetic analysis was performed using the maximum likelihood (ML) method implemented in IQ-TREE, with the optimal nucleotide substitution models selected by ModelFinder (SYM + I + R5 for the full-length genome and GTR + F + I + R4 for the Cap gene). The phylogenetic tree was constructed with 1,000 bootstrap replicates to assess branch support (13). Tree annotation was conducted using the Interactive Tree Of Life (iTOL) software,^2^ an online tool for the visualization and annotation of phylogenetic trees (14).

### Recombination analysis of the identified HLJ2024 strain

The HLJ2024 strain in this study and the reference sequences of PiCV genes from GenBank were used for the recombination analysis. The complete sequences were aligned in the ClustalX program and then screened for possible recombination using the Recombination Detection Program (RDP) (15), GENECONV (16), BootScan (17), MaxChi (18), Chimaera (19), and SiScan (20) methods embedded in RDP4. Only potential recombination events detected by five of the programs, coupled with phylogenetic evidence of recombination, were considered significant using the highest acceptable *p*-value cutoff of 0.05.

### Molecular modeling and analysis of the cap protein of PiCV

The predicted 3D structure of the PiCV Cap protein of HLJ2024 and TF4/SN/2016 (GenBank accession no. MW181928.1) was modeled using the open-source modeling server, SWISS-MODEL^3^ from the Swiss Institute of Bioinformatics. The templates in the Protein Data Bank (PDB) were selected to build a 3D structure model of the Beak and Feather Disease Virus Capsid Protein (PDB ID: 5j37.1. A). Illustrations and comparisons of these modeled tertiary structures were obtained using the Python-based molecular viewer PyMOL (The PyMOL Molecular Graphics System, Version 1.7.4 Schrdinger, LLC).

## Results

### Virus identification

**Figure 1:**
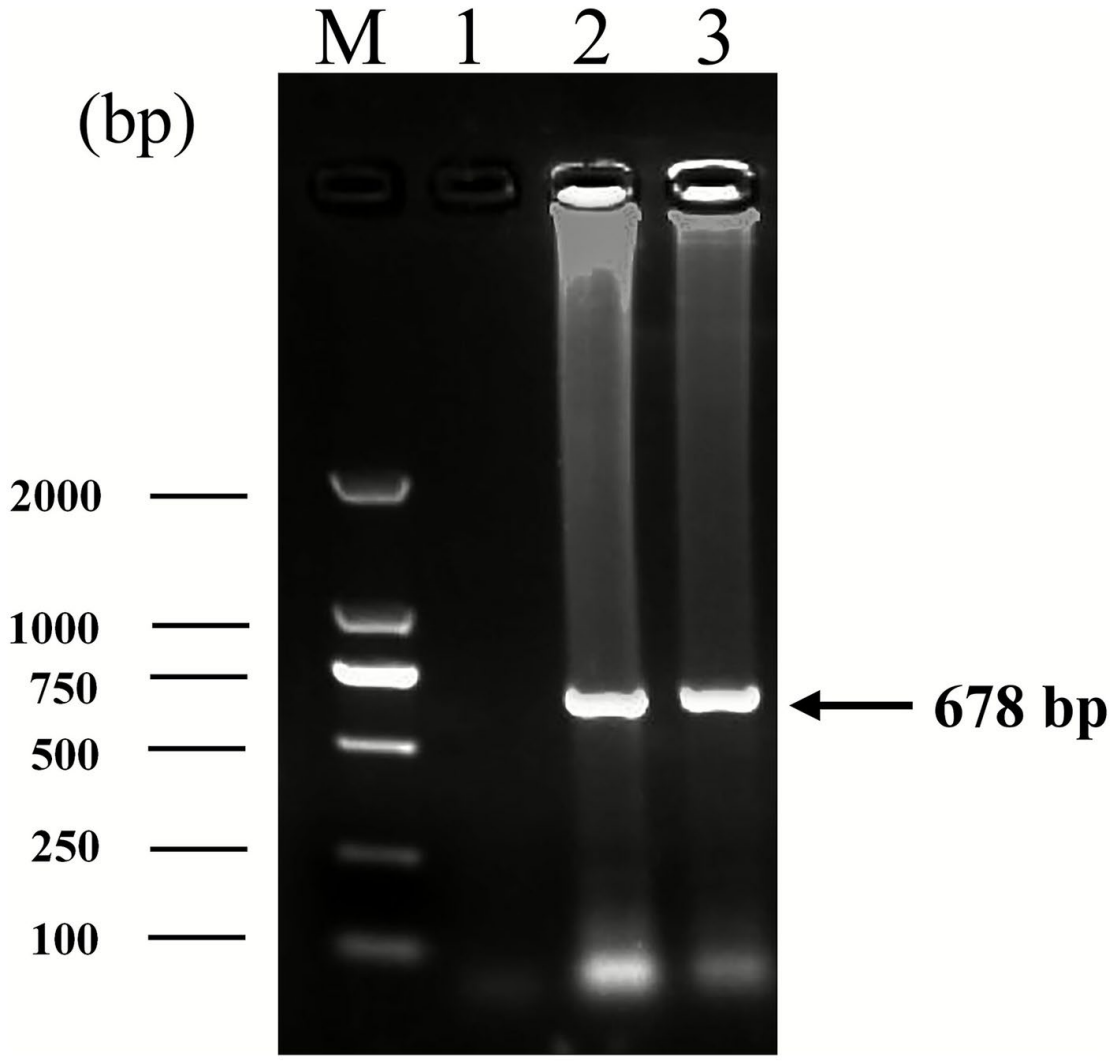
Identification of PiCV strain HLJ2024 with PCR. The extracted DNA was analyzed via PCR using PiCV-specific primers, with the gel electrophoresis results shown. Lanes are labeled as follows: Lane M, DNA molecular weight marker; Lane 1, negative control (no template DNA); Lane 2, positive control (known PiCV-positive sample); Lane 3, HLJ2024-positive sample.

To identify the HLJ2024 strain, DNA extracted from liver samples was used for PCR identification. Gene-specific PCR analysis confirmed the presence of PiCV in virus-positive liver samples (Figure 1). Additionally, tests to rule out co-infection with other viruses are provided in Supplementary Figure S1.

### Genome sequence analysis

**Figure 2:**
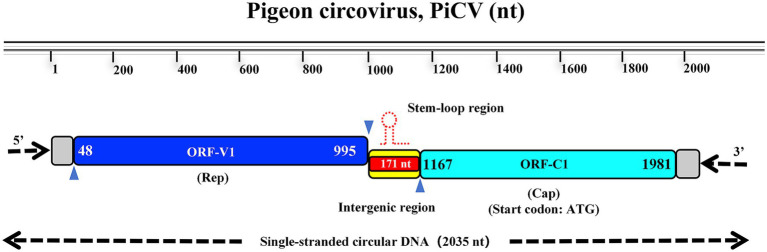
Genome structure of PiCV strain HLJ2024. PiCV strain HLJ2024 genome contains two main ORFs. ORF V1 (48–995, 948 nt) encodes Rep protein (315 aa); ORF C1 (1167–1981, 816 nt) encodes Cap protein (271 aa).

The complete genome sequence of strain PiCV HLJ2024 has been deposited in GenBank (GenBank accession no. PX496845), containing five main open reading frames (ORFs). They are ORF V1, ORF C1, ORF C2, ORF C3, and ORF C4, respectively. Among them, ORF V1 and ORF C1 are the two main coding regions. Outside the two main coding regions, there are also intergenic regions that may be related to virus replication. The length of the ORF V1 is 948 nucleotides (nt; 48–995), encoding the replication-associated protein (Rep protein) composed of 316 amino acids (aa). The length of the ORF C1 is 816 nt (1167–1981), encoding capsid proteins composed of 271 aa (Figure 2).

The nt and aa sequences of strain HLJ2024 were compared for homology with other reference strains of PiCV. A summary of pairwise nt and aa comparisons is presented in Table 1. Homology analysis revealed that strain HLJ2024 shares whole-genome, ORF V1 gene, ORF C1 gene, and ORF C1 aa sequence identities of 72.9–97.7%, 89.8–96.4%, 85.0–97.0%, and 66.9–99.3%, respectively, with other reference PiCV strains. Specifically, HLJ2024 exhibited the lowest whole-genome sequence identity (72.9%) with the Japanese strain PiCV/Japan/2/2010 (GenBank accession no. LC035390.1) from the Japan–Asia lineage, and the highest whole-genome sequence identity (97.7%) with the Chinese strain QD6/SN/2018 (GenBank accession no. MW181968.1). For the ORF V1 gene, HLJ2024 showed the lowest nt identity (89.8%) with strain SF079/ShangHai/2014 (GenBank accession no. KX108827.1) and the highest nt identity (96.4%) with the Chinese strain TF4/SN/2016 (GenBank accession no. MW181928.1).

**Table 1:** Sequence identities between the PiCV HLJ2024 strain and other reference strains of pigeon circovirus.

Virus	Nucleotide identity (%)			Amino acid identity (%)
	Genome	ORF V1	ORF C1	ORF C1
Other reference strains of PiCV	72.9–97.7	89.8–96.4	85.0–97.0	66.9–99.3

Regarding the ORF C1 gene (capsid protein, Cap), HLJ2024 displayed the lowest nt identity (85.0%) with PiCV/Japan/2/2010 (GenBank accession no. LC035390.1) and the highest nt identity (97.0%) with TF4/SN/2016 (GenBank accession no. MW181928.1). At the aa level, the ORF C1 protein of HLJ2024 shared the lowest identity (66.9%) with PiCV/Japan/2/2010 (GenBank accession no. LC035390.1) and the highest identity (99.3%) with TF4/SN/2016 (GenBank accession no. MW181928.1).

**Figure 3:**
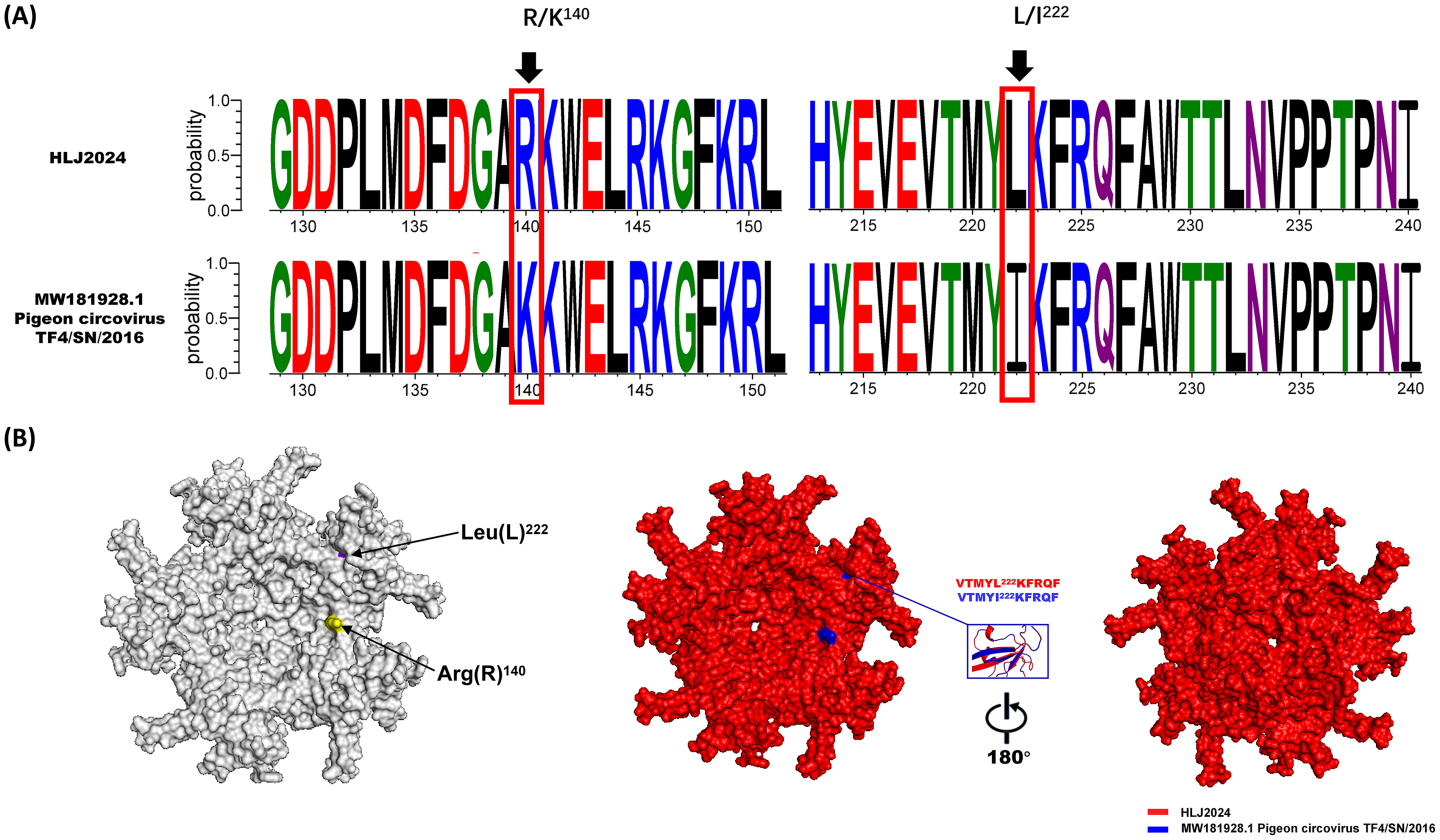
Analysis of amino acid mutation sites in the Cap protein between PiCV HLJ2024 and TF4/SN/2016 strains.‌ **(A)** Amino acid sequence alignment of the Cap protein between PiCV HLJ2024 and the reference strain TF4/SN/2016. The red boxes highlight two amino acid mutation sites in HLJ2024: lysine (K) → arginine (R) at position 144 and isoleucine (I) → leucine (L) at position 222. **(B)** Structural variations at mutation sites in the Cap protein of HLJ2024 Strain relative to TF4/SN/2016 Strain.

Further amino acid sequence alignment between HLJ2024 and the closest relative TF4/SN/2016 identified two specific mutations in the Cap protein of HLJ2024: lysine (K) → arginine (R) at position 144 and isoleucine (I) → leucine (L) at position 222 of the ORF C1 protein (Figure 3A). Comparative structural modeling analysis revealed structural differences at the 222nd amino acid residue between the two strains, suggesting potential functional implications of this mutation (Figure 3B). Electropherogram validation of these key amino acid positions is provided in Supplementary Figure S3.

### Phylogenetic analyses of HLJ2024 strain

**Figure 4:**
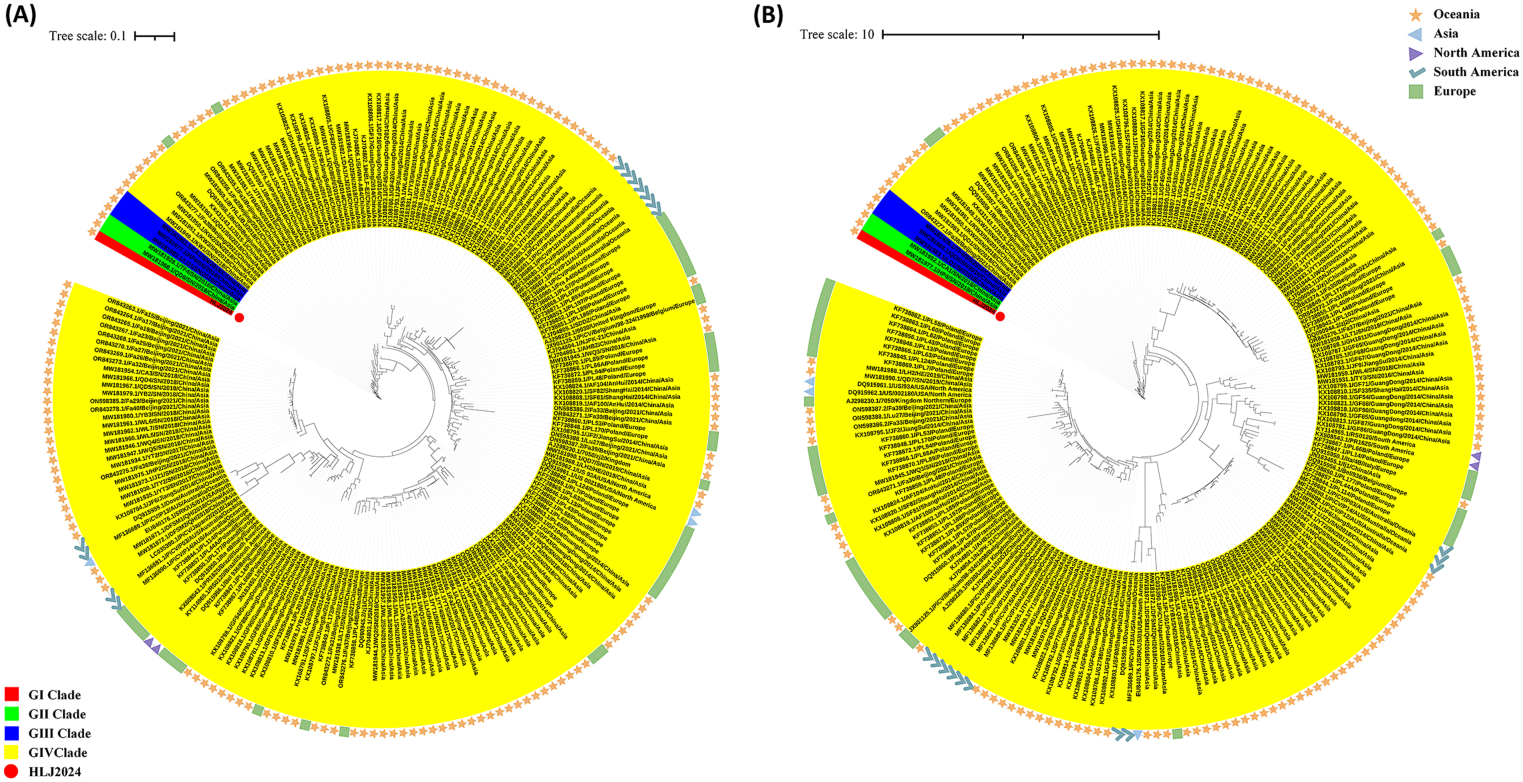
Phylogenetic analysis of PiCV strains. **(A)** Phylogenetic analysis of PiCV strains based on the whole-genome sequence. **(B)** Phylogenetic analysis of PiCV strains based on the Cap gene sequence.

Phylogenetic analysis using the maximum likelihood (ML) method implemented in IQ-TREE confirmed the evolutionary relationships between strain HLJ2024 and other reference strains of PiCV. Phylogenetic trees were constructed based on alignments of the complete genome and Cap gene nucleotide (nt) sequences (Figure 4).

Analysis of the whole-genome-based phylogenetic tree revealed that PiCV strains can be classified into four major clades (GI–GIV). Clades GI, GII, and GIII are exclusively composed of strains originating from China. In contrast, strains from Europe, Oceania, and other regions of Asia cluster within the large clade GIV, with strains from different countries or continents displaying relatively close genetic relatedness. Specifically, European strains exhibit a tight evolutionary relationship, including the Polish strain PL57 (GenBank accession no. KF738861.1) and the French strain Fra A40042 (GenBank accession no. DQ915960.1). Strains from Australia (Oceania) form a distinct subcluster and are genetically closely related to the Chinese strain QD3/SN/2018 (GenBank accession no. MW181965.1). Japanese strains (Asia) share a close evolutionary affinity with Chinese strains, such as the Japanese strain PiCV/Japan/2/2010 (GenBank accession no. LC035390.1) and the Chinese strains DFSM1/QH/2018 (GenBank accession no. MW181971.1) and DFSM2/QH/2018 (GenBank accession no. MW181972.1). The HLJ2024 strain identified in this study forms an independent, distinct new subclade based on IQ-TREE analysis and is genetically closely related to GII clade strains from China, namely TF4/SN/2016 (GenBank accession no. MW181928.1) and QD6/SN/2018 (GenBank accession no. MW181968.1; Figure 4A). Phylogenetic analysis based on the Cap gene yielded results consistent with those of the whole-genome phylogenetic tree, with congruent major clustering patterns (Figure 4B).

### Recombination analysis of HLJ2024 strain

The whole genome sequence of the HLJ2024 strain and other PiCVs from GenBank were used for the recombination analysis, sequence alignment was carried out through ClustalX program, and detection and analysis were carried out by the Recombination Detection Program (RDP) (15), GENECONV (16), BootScan (17), MaxChi (18), Chimaera (19), and SiScan (20) methods embedded in RDP4. Among them, six algorithms (RDP, GENECONV, BootScan, MaxChi, Chimaera, and SiScan) detected recombination signals in the HLJ2024 strain (all *p*-values < 0.05), with specific p-values as follows: RDP (*p* = 3.807E-15), GENECONV (*p* = 8.465E-16), BootScan (*p* = 3.964E-15), MaxChi (*p* = 6.952E-10), Chimaera (*p* = 1.460E-10), and SiScan (*p* = 1.626E-12). Relevant recombination information is presented in Supplementary Table S3.

**Figure 5:**
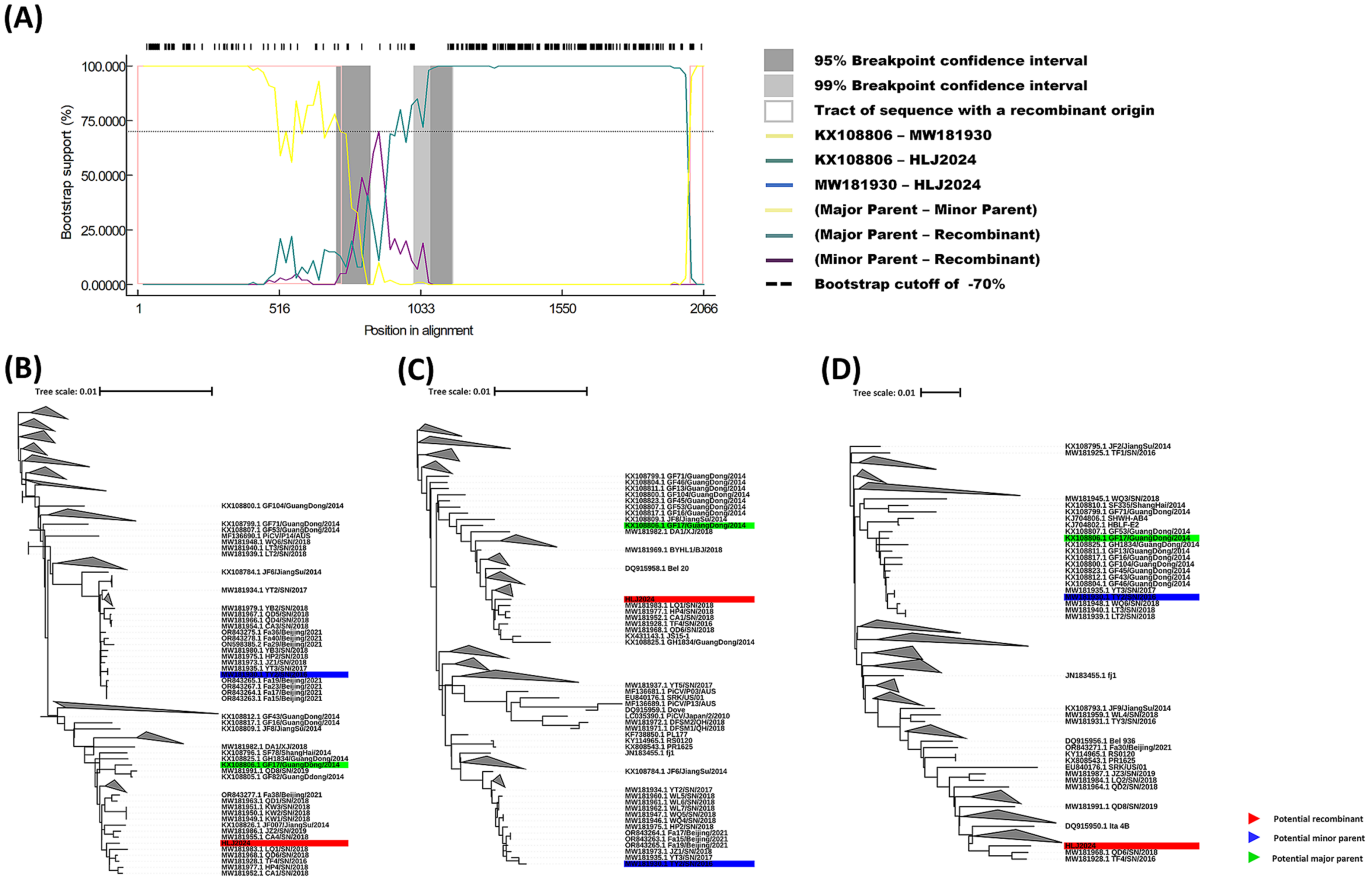
Identification of recombination events between the major parent strain, GF17/GuangDong/2014 (green), and the minor parent strain, TY2/SN/2016 (blue), which led to the recombinant HLJ2024 strain (red). **(A)** Simplot recombination analysis of three viral strains. **(B–D)** A fast neighbor-joining (NJ) tree (1,000 replicates, Kimura two-parameter distance) was constructed using the regions derived from the minor parent strain (748–2016) **(B)**, the non-recombinant region **(C)**, and recombination region (747–2017) **(D)**.

A recombination event that occurred between the GF17/GuangDong/2014 strain (GenBank accession no. KX108806) and the TY2/SN/2016 strain (GenBank accession no. MW181930) led to the generation of the recombinant HLJ2024 strain, with GF17/GuangDong/2014 as the major parent strain and TY2/SN/2016 as the minor parent strain. The recombination region was mapped to nucleotides 2017–747. The BootScan analysis map of the recombination event is presented in Figure 5A, while the other five algorithmic detection maps are displayed in Supplementary Figure S4. The recombination event was confirmed by the fast neighbor-joining trees that were constructed using the regions derived from the minor parent strain (nucleotides 747–2017; Figure 5B), the non-recombinant region (Figure 5C), and the recombinant region (nucleotides 2017–747; Figure 5D).

## Discussion

This study is the first to characterize the molecular evolutionary features of the PiCV strain HLJ2024 isolated from Heilongjiang Province, China. Whole-genome homology analysis revealed that HLJ2024 shared the highest nucleotide identity (97.7%) with the Chinese strain QD6/SN/2018 (GenBank accession no. MW181968.1). At the amino acid level, the Cap protein of HLJ2024 shared the highest identity (99.3%) with the Chinese strain TF4/SN/2016, far exceeding its identity with strains from other regions. This result confirms the presence of distinct geographical clustering of PiCV strains within China, which is consistent with the findings of nationwide epidemiological investigations reported by Xing et al. (21). Notably, phylogenetic analysis based on a tree constructed using IQ-TREE demonstrated that the HLJ2024 strain formed an independent subclade. Within the phylogenetic topology, HLJ2024 showed the closest genetic relationship with the Chinese strains TF4/SN/2016 (GenBank accession no. MW181928.1) and QD6/SN/2018 (GenBank accession no. MW181968.1), which is congruent with the results of the homology analysis. Regarding clade GIV, although strains derived from different continents were interspersed within this clade, strains from distinct countries still displayed relatively close genetic affinities. Consistent with previous reports, the migration and transboundary trade (import/export) of racing pigeons are proposed to be key pathways facilitating cross-regional viral gene introgression (22–24). A key limitation of this study is that it was based on a single PiCV isolate (HLJ2024). Therefore, the conclusions regarding the evolutionary trends of PiCV should be further verified using a larger sample size encompassing strains from more diverse geographical regions—this will help enhance the generalizability and robustness of the findings.

Further amino acid sequence alignment between HLJ2024 and its closest relative TF4/SN/2016 identified two specific mutations in the ORF C1 protein of HLJ2024: a lysine (K) → arginine (R) substitution at position 144 and an isoleucine (I) → leucine (L) substitution at position 222. Notably, comparative structural modeling analysis revealed distinct structural differences at the 222nd amino acid residue between the two strains, suggesting potential functional implications of this mutation. To validate the accuracy of these mutations and address potential sequencing errors inherent to next-generation sequencing (NGS), we performed PCR-Sanger sequencing (the gold standard for sequence verification) targeting the ORF C1 gene of HLJ2024. Sanger sequencing electropherograms showed clear, single peaks at positions 144 and 222, confirming the R^144^K and L^222^I substitutions. Notably, this verification process identified discrepancies between the initial NGS-derived sequence and the Sanger-confirmed sequence—specifically, the NGS data initially predicted a non-existent mutation at position 201 (K → N), which was corrected following Sanger validation. Such discrepancies are not uncommon in NGS-based genome assembly, particularly for viral genomes with high GC content or repetitive regions, where base-calling algorithms may generate false-positive mutations due to low coverage depth or sequencing bias. At the molecular level, the R^144^-K mutation involves a substitution of two positively charged amino acids (arginine and lysine), which may preserve local electrostatic properties but could still modulate protein–protein interactions—consistent with findings that even conservative charge-preserving mutations in circovirus proteins can alter binding affinity to host factors. More notably, the L^222^-I mutation replaces leucine with isoleucine, two hydrophobic amino acids with differing side-chain branching. Such structural variations in hydrophobic core residues have been shown to impact protein folding dynamics and stability in circoviruses, which may further affect the ORF C1 protein’s biological function (25). Studies on porcine circovirus type 2 (PCV2) have established that mutations in key functional regions of viral proteins can mediate critical phenotypic alterations, such as immune escape or altered tissue tropism. For instance, mutations in the loop structures of the PCV2 Cap protein (which are homologous to potential functional domains in PiCV ORF C1) can disrupt virus–host interactions and alter *in vitro* replication efficiency (26, 27). While the specific role of PiCV ORF Cap protein remains to be fully elucidated, its homology to circovirus functional proteins suggests that mutations at positions 144 and 222 could modulate host–pathogen interactions. If these residues localize to functional domains, their substitutions might induce cooperative conformational changes that alter protein stability or binding activity—though this requires validation through high-resolution structural studies. Mutations at analogous positions to 144 and 222 have been detected in HLJ2024-proximal strains, implying these sites may be subject to potential evolutionary selection, though no population genetics analysis, such as dN/dS, was performed to confirm this. Thus, long-term genomic surveillance of clinical PiCV isolates is imperative to track the prevalence of these mutations and evaluate their potential association with phenotypic changes and vaccine pressure.

In conclusion, this study determined the complete genome sequence of PiCV strain HLJ2024. Recombination analysis indicated its origin from the recombination of two Chinese indigenous strains, while phylogenetic analysis revealed its independent subclade status and close genetic relationship with Chinese PiCV lineages. A key I^222^L mutation (isoleucine→leucine) was identified in the Cap protein. These findings establish a molecular basis for exploring PiCV’s genetic evolution mechanisms, particularly in Northeast China. The detection of mutations and recombination in HLJ2024 underscores PiCV’s potential for continuous evolutionary changes, highlighting the imperative of systematic evolutionary surveillance.

## Data Availability

The data presented in this study are deposited in public repositories. The complete genome sequence is available in the GenBank repository under accession number PX496845. The associated raw sequencing reads are available in the NCBI Sequence Read Archive (SRA) under BioProject accession number PRJNA1370006 and SRA run accession number SRR36222660.

## Footnotes

1 https://blast.ncbi.nlm.nih.gov/Blast.cgi

2 http://itol.embl.de/

3 https://swissmodel.expasy.org/

## Supplementary material

SUPPLEMENTARY FIGURE S1 Detection of Newcastle disease virus (NDV) to rule out co-infection in samples. This figure shows the results of NDV-specific PCR for excluding NDV co-infection in the samples, with gel electrophoresis results presented. Lanes are labeled as follows: Lane M, DNA molecular weight marker; Lane 1, negative control; Lane 2, PiCV-positive control; Lane 3, test sample (HLJ2024-related sample); Lane 4, negative control; Lane 5, NDV-positive control; Lane 6, test sample (HLJ2024-duplicate). A specific amplification band corresponding to NDV is only observed in Lane 5 (NDV-positive control), while no target band is detected in Lanes 4 and 6.

SUPPLEMENTARY FIGURE S2 Agarose gel electrophoresis identification of PCR-amplified products covering the full-length genome of PiCV. Agarose gel electrophoresis analysis of PCR-amplified products covering the full-length genome of PiCV. Lanes 1-2: PCR-amplified fragment with a length of 1961 bp; Lanes 3-4: PCR-amplified fragment with a length of 1265 bp; Lanes 5-6: PCR-amplified fragment with a length of 1922 bp; Lane 7: Negative control.

SUPPLEMENTARY FIGURE S3 Sanger sequencing electropherograms of the novel amino acid mutation sites (positions 144 and 222) in the Cap gene of PiCV HLJ2024. Sanger sequencing validation of the two novel amino acid mutation sites identified in the Cap gene of PiCV HLJ2024. **(A)** Electropherogram of the K→R mutation at position 144 of the Cap gene. **(B)** Electropherogram of the I→L mutation at position 222 of the Cap gene; the red box indicates the specific nucleotide variation responsible for the amino acid change.

SUPPLEMENTARY FIGURE S4 Validation maps of recombination signals in PiCV strain HLJ2024 (RDP, GENECONV, MaxChi, SiScan, and Chimaera). Recombination signals of PiCV strain HLJ2024 were verified via five algorithms in RDP4 software, with corresponding maps shown in **(A–E)**. **(A)** RDP map: Curve shows recombination probability at each position, with a significant peak at the breakpoint (*p* = 3.807E-15, *p* < 0.05), indicating high confidence in recombination. **(B)** GENECONV map: Similarity curve/heatmap displays homologous exchange intensity between HLJ2024 and parental strains (*p* = 8.465E-16, *p* < 0.05), confirming the occurrence of homologous exchange. **(C)** MaxChi map: Statistical score curve shows a sharp peak at the breakpoint (*p* = 6.952E-10, *p* < 0.05), supporting the presence of recombination. **(D)** SiScan map: Sliding-window similarity curve shows a sharp score jump at the breakpoint (*p* = 1.626E-12, *p* < 0.05), verifying the recombination event. **(E)** Chimaera map: Signal intensity graph presents a significant peak at the breakpoint (*p* = 1.460E-10, *p* < 0.05), further confirming recombination.
