# Supplementary material for: Whole-genome characterization and phylogenetic analysis of pigeon circovirus in racing pigeons from Heilongjiang, China
Source: Front Vet Sci. 2026 Jan 5;12:1685178. doi: 10.3389/fvets.2025.1685178 (PMC12812675; doi:10.3389/fvets.2025.1685178)
Supplement: Supplementary file 1 [file Table_1.docx]

| **Primer name** | **Sequence (5’-3’)** | **Target** | **Reference** |
| --- | --- | --- | --- |
| PiCV-F | GGAGCCACGGAGCCACAT | 1922 bp | This study |
| PiCV-R1 | CGGATCAGGAGACGAAGGACAC |  |  |
| PiCV-F | GGAGCCACGGAGCCACAT | 1961 bp |  |
| PiCV-R2 | CCCCCGACCCCCAACAT |  |  |
| PiCV-F | GGAGCCACGGAGCCACAT | 1265 bp |  |
| PiCV-R3 | CGGAGATTCAGACGCAGGAG |  |  |

**Table S1.** Primers used for amplification of the full-length genome sequence.
