# Supplementary material for: Whole-genome characterization and phylogenetic analysis of pigeon circovirus in racing pigeons from Heilongjiang, China
Source: Front Vet Sci. 2026 Jan 5;12:1685178. doi: 10.3389/fvets.2025.1685178 (PMC12812675; doi:10.3389/fvets.2025.1685178)
Supplement: Supplementary file 2 [file Table_2.docx]

| **Table S2.**  The reference pigeon circovirus strains used in this study. | | | |
| --- | --- | --- | --- |
| **GenBank accession no.** | **Strains name** | **Coutry** | **Continent** |
| AJ298229.1 | 9030 | United Kingdom:Northern Ireland" | Europe |
| AJ298230.1 | 7050 | United Kingdom:Northern Ireland | Europe |
| DQ090944.2 | zj2 | China | Asia |
| DQ090945.2 | zj1 | China | Asia |
| DQ915950.1 | Ita 4B | Italy | Europe |
| DQ915956.1 | Bel 936 | Belgium | Europe |
| DQ915957.1 | Bel 18 | Belgium | Europe |
| DQ915958.1 | Bel 20 | Belgium | Europe |
| DQ915959.1 | Dove | Australia | Oceania |
| DQ915960.1 | Fra A40042 | France | Europe |
| DQ915961.1 | US 93A | USA | North America |
| DQ915962.1 | US 002180 | USA | North America |
| EU840176.1 | SRK/US/01 | USA | North America |
| JN183455.1 | fj1 | China | Asia |
| JX901125.1 | PiCV/Belgium/98-324/1998 | Belgium | Europe |
| KF738843.1 | PL102 | Poland | Europe |
| KF738844.1 | PL114 | Poland | Europe |
| KF738845.1 | PL124 | Poland | Europe |
| KF738846.1 | PL13 | Poland | Europe |
| KF738847.1 | PL14 | Poland | Europe |
| KF738848.1 | PL170 | Poland | Europe |
| KF738849.1 | PL172 | Poland | Europe |
| KF738850.1 | PL177 | Poland | Europe |
| KF738851.1 | PL188 | Poland | Europe |
| KF738852.1 | PL189 | Poland | Europe |
| KF738853.1 | PL197 | Poland | Europe |
| KF738854.1 | PL201 | Poland | Europe |
| KF738855.1 | PL40 | Poland | Europe |
| KF738856.1 | PL43 | Poland | Europe |
| KF738857.1 | PL44A | Poland | Europe |
| KF738858.1 | PL44B | Poland | Europe |
| KF738859.1 | PL48 | Poland | Europe |
| KF738860.1 | PL53 | Poland | Europe |
| KF738861.1 | PL57 | Poland | Europe |
| KF738862.1 | PL58 | Poland | Europe |
| KF738863.1 | PL60 | Poland | Europe |
| KF738864.1 | PL62 | Poland | Europe |
| KF738865.1 | PL63 | Poland | Europe |
| KF738866.1 | PL66A | Poland | Europe |
| KF738867.1 | PL66B | Poland | Europe |
| KF738868.1 | PL67 | Poland | Europe |
| KF738869.1 | PL7 | Poland | Europe |
| KF738870.1 | PL89 | Poland | Europe |
| KF738871.1 | PL89X | Poland | Europe |
| KF738872.1 | PL94 | Poland | Europe |
| KJ704801.1 | AHBZ | China | Asia |
| KJ704802.1 | HBLF-E2 | China | Asia |
| KJ704803.1 | JSNJ | China | Asia |
| KJ704804.1 | NJPK-21 | China | Asia |
| KJ704805.1 | SDDZ | China | Asia |
| KJ704806.1 | SHWH-AB4 | China | Asia |
| KX108780.1 | GF42/GuangDong/2014 | China | Asia |
| KX108781.1 | GF86/GuangDong/2014 | China | Asia |
| KX108782.1 | SF77/ShangHai/2014 | China | Asia |
| KX108783.1 | GF67/GuangDong/2014 | China | Asia |
| KX108784.1 | JF6/JiangSu/2014 | China | Asia |
| KX108785.1 | GF68/GuangDong/2014 | China | Asia |
| KX108786.1 | G2798/GuangDong/2014 | China | Asia |
| KX108787.1 | GF69/GuangDong/2014 | China | Asia |
| KX108788.1 | GH1811/GuangDong/2014 | China | Asia |
| KX108790.1 | GF85/Guangdong/2014 | China | Asia |
| KX108791.1 | SF76/ShangHai/2014 | China | Asia |
| KX108792.1 | GF103/Guangdong/2014 | China | Asia |
| KX108793.1 | JF9/JiangSu/2014 | China | Asia |
| KX108794.1 | SF85/Shanghai/2014 | China | Asia |
| KX108795.1 | JF2/JiangSu/2014 | China | Asia |
| KX108796.1 | SF78/ShangHai/2014 | China | Asia |
| KX108797.1 | JF3/JiangSu/2014 | China | Asia |
| KX108798.1 | GF54/GuangDong/2014 | China | Asia |
| KX108799.1 | GF71/GuangDong/2014 | China | Asia |
| KX108800.1 | GF104/GuangDong/2014 | China | Asia |
| KX108801.1 | JF45/JiangSu/2014 | China | Asia |
| KX108802.1 | GF81/GuangDong/2014 | China | Asia |
| KX108803.1 | SF80/GuangDong/2014 | China | Asia |
| KX108804.1 | GF46/GuangDong/2014 | China | Asia |
| KX108805.1 | GF82/GuangDdong/2014 | China | Asia |
| KX108806.1 | GF17/GuangDong/2014 | China | Asia |
| KX108807.1 | GF53/GuangDong/2014 | China | Asia |
| KX108808.1 | SF81/ShangHai/2014 | China | Asia |
| KX108809.1 | JF8/JiangSu/2014 | China | Asia |
| KX108810.1 | SF335/ShangHai/2014 | China | Asia |
| KX108812.1 | GF43/GuangDong/2014 | China | Asia |
| KX108813.1 | GF87/GuangDong/2014 | China | Asia |
| KX108814.1 | SF86/ShangHai/2014 | China | Asia |
| KX108815.1 | GF84/GuangDong/2014 | China | Asia |
| KX108817.1 | GF16/GuangDong/2014 | China | Asia |
| KX108818.1 | GF90/GuangDong/2014 | China | Asia |
| KX108819.1 | AF100/AnHui/2014 | China | Asia |
| KX108820.1 | SF82/ShangHai/2014 | China | Asia |
| KX108821.1 | GF88/GuangDong/2014 | China | Asia |
| KX108822.1 | SF83/ShangHai/2014 | China | Asia |
| KX108823.1 | GF45/GuangDong/2014 | China | Asia |
| KX108824.1 | AF104/AnHui/2014 | China | Asia |
| KX108825.1 | GH1834/GuangDong/2014 | China | Asia |
| KX108826.1 | JF007/JiangSu/2014 | China | Asia |
| KX108827.1 | SF079/ShangHai/2014 | China | Asia |
| KX431143.1 | JS15-1 | China | Asia |
| KX808543.1 | PR1625 | Brazil | South America |
| KY114965.1 | RS0120 | Brazil | South America |
| LC035390.1 | PiCV/Japan/2/2010 | Japan: Kagawa | Asia |
| MF136680.1 | PiCV/P02/AUS | Australia | Oceania |
| MF136681.1 | PiCV/P03/AUS | Australia | Oceania |
| MF136682.1 | PiCV/P05/AUS | Australia | Oceania |
| MF136684.1 | PiCV/P08/AUS | Australia | Oceania |
| MF136686.1 | PiCV/P10/AUS | Australia | Oceania |
| MF136687.1 | PiCV/P11/AUS | Australia | Oceania |
| MF136688.1 | PiCV/P12/AUS | Australia | Oceania |
| MF136689.1 | PiCV/P13/AUS | Australia | Oceania |
| MF136690.1 | PiCV/P14/AUS | Australia | Oceania |
| MF136691.1 | PiCV/P15/AUS | Australia | Oceania |
| MW181925.1 | TF1/SN/2016 | China | Asia |
| MW181926.1 | TF2/SN/2016 | China | Asia |
| MW181927.1 | TF3/SN/2016 | China | Asia |
| MW181928.1 | TF4/SN/2016 | China | Asia |
| MW181929.1 | TY1/SN/2016 | China | Asia |
| MW181930.1 | TY2/SN/2016 | China | Asia |
| MW181931.1 | TY3/SN/2016 | China | Asia |
| MW181932.1 | SX1/SN/2017 | China | Asia |
| MW181933.1 | YT1/SN/2017 | China | Asia |
| MW181934.1 | YT2/SN/2017 | China | Asia |
| MW181935.1 | YT3/SN/2017 | China | Asia |
| MW181936.1 | YT4/SN/2017 | China | Asia |
| MW181937.1 | YT5/SN/2017 | China | Asia |
| MW181939.1 | LT2/SN/2018 | China | Asia |
| MW181940.1 | LT3/SN/2018 | China | Asia |
| MW181941.1 | LT4/SN/2018 | China | Asia |
| MW181942.1 | LT5/SN/2018 | China | Asia |
| MW181943.1 | WQ1/SN/2018 | China | Asia |
| MW181944.1 | WQ2/SN/2018 | China | Asia |
| MW181945.1 | WQ3/SN/2018 | China | Asia |
| MW181946.1 | WQ4/SN/2018 | China | Asia |
| MW181947.1 | WQ5/SN/2018 | China | Asia |
| MW181948.1 | WQ6/SN/2018 | China | Asia |
| MW181949.1 | KW1/SN/2018 | China | Asia |
| MW181950.1 | KW2/SN/2018 | China | Asia |
| MW181951.1 | KW3/SN/2018 | China | Asia |
| MW181952.1 | CA1/SN/2018 | China | Asia |
| MW181953.1 | CA2/SN/2018 | China | Asia |
| MW181954.1 | CA3/SN/2018 | China | Asia |
| MW181955.1 | CA4/SN/2018 | China | Asia |
| MW181956.1 | WL1/SN/2018 | China | Asia |
| MW181957.1 | WL2/SN/2018 | China | Asia |
| MW181958.1 | WL3/SN/2018 | China | Asia |
| MW181959.1 | WL4/SN/2018 | China | Asia |
| MW181960.1 | WL5/SN/2018 | China | Asia |
| MW181961.1 | WL6/SN/2018 | China | Asia |
| MW181962.1 | WL7/SN/2018 | China | Asia |
| MW181963.1 | QD1/SN/2018 | China | Asia |
| MW181964.1 | QD2/SN/2018 | China | Asia |
| MW181965.1 | QD3/SN/2018 | China | Asia |
| MW181966.1 | QD4/SN/2018 | China | Asia |
| MW181967.1 | QD5/SN/2018 | China | Asia |
| MW181968.1 | QD6/SN/2018 | China | Asia |
| MW181969.1 | BYHL1/BJ/2018 | China | Asia |
| MW181970.1 | DS1/GS/2018 | China | Asia |
| MW181971.1 | DFSM1/QH/2018 | China | Asia |
| MW181972.1 | DFSM2/QH/2018 | China | Asia |
| MW181973.1 | JZ1/SN/2018 | China | Asia |
| MW181974.1 | HP1/SN/2018 | China | Asia |
| MW181975.1 | HP2/SN/2018 | China | Asia |
| MW181976.1 | HP3/SN/2018 | China | Asia |
| MW181977.1 | HP4/SN/2018 | China | Asia |
| MW181978.1 | YB1/SN/2018 | China | Asia |
| MW181979.1 | YB2/SN/2018 | China | Asia |
| MW181980.1 | YB3/SN/2018 | China | Asia |
| MW181981.1 | YB4/SN/2018 | China | Asia |
| MW181982.1 | DA1/XJ/2018 | China | Asia |
| MW181983.1 | LQ1/SN/2018 | China | Asia |
| MW181984.1 | LQ2/SN/2018 | China | Asia |
| MW181985.1 | LQ3/SN/2018 | China | Asia |
| MW181986.1 | JZ2/SN/2019 | China | Asia |
| MW181987.1 | JZ3/SN/2019 | China | Asia |
| MW181988.1 | LH2/HE/2019 | China | Asia |
| MW181989.1 | LH3/HE/2019 | China | Asia |
| MW181990.1 | QD7/SN/2019 | China | Asia |
| MW181991.1 | QD8/SN/2019 | China | Asia |
| ON598385.2 | Fa29/Beijing/2021 | China | Asia |
| ON598386.2 | Fa33/Beijing/2021 | China | Asia |
| ON598387.2 | Fa39/Beijing/2021 | China | Asia |
| ON598388.1 | Lu27/Beijing/2021 | China | Asia |
| OR843255.1 | Fa1/Beijing/2021 | China | Asia |
| OR843256.1 | Fa2/Beijing/2021 | China | Asia |
| OR843257.1 | Fa3/Beijing/2021 | China | Asia |
| OR843258.1 | Fa4/Beijing/2021 | China | Asia |
| OR843259.1 | Fa5/Beijing/2021 | China | Asia |
| OR843260.1 | Fa6/Beijing/2021 | China | Asia |
| OR843261.1 | Fa8/Beijing/2021 | China | Asia |
| OR843262.1 | Fa11/Beijing/2021 | China | Asia |
| OR843263.1 | Fa15/Beijing/2021 | China | Asia |
| OR843264.1 | Fa17/Beijing/2021 | China | Asia |
| OR843265.1 | Fa19/Beijing/2021 | China | Asia |
| OR843266.1 | Fa21/Beijing/2021 | China | Asia |
| OR843267.1 | Fa23/Beijing/2021 | China | Asia |
| OR843268.1 | Fa25/Beijing/2021 | China | Asia |
| OR843269.1 | Fa26/Beijing/2021 | China | Asia |
| OR843270.1 | Fa26/Beijing/2021 | China | Asia |
| OR843271. | Fa30/Beijing/2021 | China | Asia |
| OR843272.1 | Fa31/Beijing/2021 | China | Asia |
| OR843273.1 | Fa32/Beijing/2021 | China | Asia |
| OR843274.1 | Fa35/Beijing/2021 | China | Asia |
| OR843275.1 | Fa36/Beijing/2021 | China | Asia |
| OR843276.1 | Fa37/Beijing/2021 | China | Asia |
| OR843277.1 | Fa38/Beijing/2021 | China | Asia |
| OR843278.1 | Fa40/Beijing/2021 | China | Asia |
