# Supplementary material for: Whole-genome characterization and phylogenetic analysis of pigeon circovirus in racing pigeons from Heilongjiang, China
Source: Front Vet Sci. 2026 Jan 5;12:1685178. doi: 10.3389/fvets.2025.1685178 (PMC12812675; doi:10.3389/fvets.2025.1685178)
Supplement: Supplementary file 3 [file Table_3.doc]

**Table S3. The breakpoint positions of recombination events in our study.**

| Breakpoint Positions in Recombinant Sequence | | | | | Detection Methods | | | | | | | | |
| --- | --- | --- | --- | --- | --- | --- | --- | --- | --- | --- | --- | --- | --- |
| Begin | End | Recombinant Sequence | Minor Parental Sequence | Major Parental Sequence | RDP | GENECONV | Bootscan | Maxchi | Chimaera | SiSscan | PhylPro | LARD | 3Seq |
| 2017 | 747 | HLJ2024 | MW181930.1_Pigeon_circovirus_TY2/SN/2016) | KX108806.1_Pigeon_circovirus_GF17/GuangDong/2014 | 3.807*10^-15^ | 8.465*10^-16^ | 3.964*10^-15^ | 6.952*10^-10^ | 1.460*10^-10^ | 1.626*10^-12^ | -- | -- | -- |

***Note****.* Minor Parent = Parent contributing the smaller fraction of sequence. Major Parent = Parent contributing the larger fraction of sequence. NS = No significant *P*-value was recorded for this recombination event using this method.
